# Case Report: Unusual Clinical Presentation of a Rare Cardiac Inflammatory Myofibroblastic Tumor in Children: The Differential Diagnosis With Pediatric Emergencies

**DOI:** 10.3389/fped.2021.718157

**Published:** 2021-11-10

**Authors:** Clio Bilotta, Giulio Perrone, Emiliano Maresi, Giovanni De Lisi, Pietro Di Pasquale, Ettore Piro, Antonina Argo, Stefania Zerbo

**Affiliations:** ^1^Department of Health Promotion, Mother and Child Care, Internal Medicine and Medical Specialties, Section of Legal Medicine, University of Palermo, Palermo, Italy; ^2^Division of Cardiology, Paolo Borsellino, G. F. Ingrassia Hospital, Palermo, Italy; ^3^Department of Health Promotion, Maternal and Child Care, Internal Medicine and Medical Specialties “G. D'Alessandro”, Neonatal Intensive Care Unit, University of Palermo, Palermo, Italy

**Keywords:** inflammatory myofibroblastic tumor, heart, children, rare tumor, immunohistochemical investigations

## Abstract

**Introduction:** There are still no guidelines about pediatric cardiac cancers. The purpose of this work is to provide new scientific data facilitating the differential diagnosis of a rare cardiac tumor with an unusual presentation, such as the cardiac inflammatory myofibroblastic tumor (IMT).

**Case Presentation:** A 3-year-old male child presented with several symptoms including unconsciousness, vomiting, and drowsiness. A clinical and neurological examination revealed a unilateral (right) motor delay and positive unilateral Babinski sign. Electrocardiogram (ECG) was normal.

**Diagnostic Assessment:** The total body computed tomography (CT) scans showed hypodensity in the left temporal–parietal lobe, a large hypodense area in the right frontal lobe, and a second area in the left frontal lobe were found with head CT. A magnetic resonance (MR) also noted cerebral areas of hypointensity. The echocardiographic images revealed an ovoid mass, adherent to the anterolateral papillary muscle. The histological exams, performed with hematoxylin–eosin, Masson's trichrome, Alcian blue PAS, Weigert and Van-Gieson stain, allowed observing the microscopic structure of the neoplastic mass. The immunohistochemical analysis was performed through subsequent antibodies: anti-vimentin, anti-actina, anti-ALK, anti-CD8, anti-CD3, anti-CD20, anti-kappa and lambda chains, and anti CD68 antibodies. The healthcare professionals diagnosed a cardiac IMT with brain embolism.

**Differential Diagnosis:** The ventricular localization, observed through radiological exams, required a differential diagnosis with fibroma and rhabdomyoma, the presence of brain embolism with sarcoma, and its morphology with fibroma. Neurological symptoms might be attributed to encephalitis, primitive cerebral cancer, such as astrocytoma or neuroblastoma, cerebral metastases due to any malignancy, or embolic stroke.

**Conclusion:** New studies are encouraged to better define IMT behavior and draw up guidelines confirming the crucial role of multidisciplinary approach and treatment protocol selected on the basis of the characteristics of the tumors, in the case of this rare type of cancer.

## Introduction

The cardiac inflammatory myofibroblastic tumor (IMT) represents a rare form of parenchymal organs and/or soft tissue sarcoma, originating from the lining of the airways, gastrointestinal tract, and reproductive system, as well as from the central nervous system. It can also involve the mesentery (1, 2).

As the name suggests, this tumor originates from mesenchymal cells, especially from myofibroblasts. The adjective “inflammatory” refers to the histopathological characterization of this tumor, marked by the presence of immune cells, among which plasma cells, lymphocytes, and eosinophils surrounding elongated myofibroblasts (3). The inflammatory process was triggered by NFkB production (4).

This disease affects most children and young adults with regard to the age of involved patients, although some cases relate to elderly subjects.

Heart involvement is quite rare: ventricular chambers represent the most affected site, and there was no different prevalence between the two ventricles (5).

Macroscopically, the tumor appears as a single or multilobulated mass, ranging in size from 1.0 to 22 cm (6).

Signs and symptoms are initially unspecified: fever, weight loss, and asthenia are associated with laboratory abnormalities, especially hypochromic and microcytic anemia, thrombocytosis, increased erythrocyte sedimentation rate, and elevated C-reactive protein (7, 8).

The clinical picture of heart involvement includes dyspnea, tachycardia, and heart failure; signs and symptoms vary based on the organ affected. The mortality of the IMT, like other cardiac tumors, is secondary to onset of arrhythmic cardiac events, thromboembolism, and outflow or inflow tract ventricular obstruction (5).

We introduce an unusual clinical presentation of a cardiac inflammatory myofibroblastic tumor (IMT) in a child. There are still no guidelines about pediatric cardiac cancers. The purpose of this work is to provide new scientific data facilitating the differential diagnosis of a rare cardiac neoplasm with an unusual presentation, such as the cardiac inflammatory myofibroblastic tumor (IMT). This study encourages the drafting of guidelines about the multidisciplinary approach in the differential diagnosis of cardiac tumors and treatment protocol selected on the basis of the characteristics of the tumors.

## Case Description

A well-being infant of 3 years old, without a relevant past medical history, was admitted to the pediatric emergency department with several symptoms, such as unconsciousness, vomiting, and drowsiness. Right motor delay and positive Babinski sign developed after a few hours; healthcare workers, assuming a form of encephalitis, treated the infant with antibiotic, antiviral, and antiedema therapy, after an infectious disease counseling. Electrocardiogram (ECG) was normal; a brain CT (computed tomography) showed a region of hypodensity in the left parietal–temporal area. A brain magnetic resonance (MR) raised the suspicion of a cerebral embolism. There was a radiological worsening, characterized by an extent of hypodensity at the left frontal–temporal lobe and homolateral lenticular–capsular region, and associated with a clinical worsening, denoted by onset of aphasia, miosis, deviation of the eyes, and upper limb hypertonia, on the third day. The echocardiographic images showed an abnormal mass inside the left ventricular chamber, on the 5th day; thus, an endocavitary thrombus was supposed, and healthcare workers administered a heparin treatment and transferred the child to the pediatric cardiac surgery department. The chest CT revealed an expansive neoformation, on the following day; the clinical picture worsened further, with onset of a comatose state, generalized tonic–clonic seizures, upper limb dystonia, requiring an intubation and mechanical ventilation, on the same day. The following brain CT showed multifocal cerebral ischemia involving also the right frontal lobe in association with periencephalic liquor space flattening, on the 9th day. The cardiac tumoral mass was surgically excised, on the 10th day. The infant, presenting persistent clinical symptoms of the previous brain embolism, such as aphasia, conjugate eye left deviation, facio-brachio-crural right hemiparesis, and upper limb ipsilateral hypertonia, was transferred from the surgical ward to a rehabilitation center 12 days after the surgery.

The following histological exams revealed the presence of a cardiac inflammatory myofibroblastic tumor (IMT). The resection of the tumor was complete. The healthcare workers avoided chemotherapy or radiotherapy according to the benign nature of the tumor and disputes in the literature concerning the adjuvant treatment with chemotherapy or radiotherapy. The anticoagulation therapy allowed the resolution of brain embolism.

The 2-year follow-up includes echocardiography, chest and brain CT, and neurological examinations. The surgical approach was sufficient in this case; in fact, the last follow-up excluded a cardiac tumor recurrence and revealed a complete resolution of brain embolism. Rehabilitation improved upper limb motility and aphasia. Conjugate eye left deviation is unchanged.

## Diagnostic Assessment

**Figure 1:**
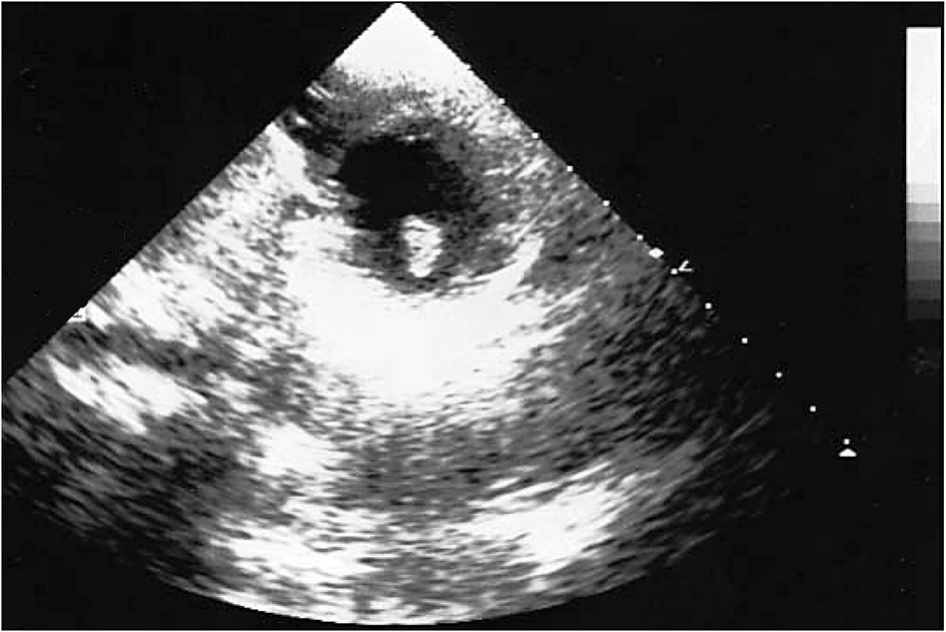
Echocardiographic image: solid neoplasm floating in the left ventricle outflow chamber, connected to the anterolateral papillary muscle of the left ventricle and characterized by homogeneous density, large base implant, and regular margins.

The brain CT scans showed hypodensity in the left temporal–parietal lobe. The follow-up, which performed head CT, highlighted a significant increase in brain damage after 3 days of hospitalization, with the involvement of unilateral basal ganglia and internal capsule. The healthcare professionals suspected an encephalitis; a further diagnostic in-depth investigation run with magnetic resonance (MR) also noted cerebral areas of hypointensity. The suspected diagnosis was brain embolism. A further brain CT highlighted a large hypodense area in the right frontal lobe and a second area in the left frontal lobe, secondary to the multi-infarct vascular lesions, with an alteration in the volume of liquor spaces. The echocardiographic images (Figure 1) revealed an ovoid mass, adherent to the anterolateral papillary muscle and characterized by a similar echogenicity to the myocardial tissue with a mobile offshoot located in the outflow tract; the radiologists confirmed a suspicion of a single expansionary lesion through CT scan of the chest with contrast.

**Figure 2:**
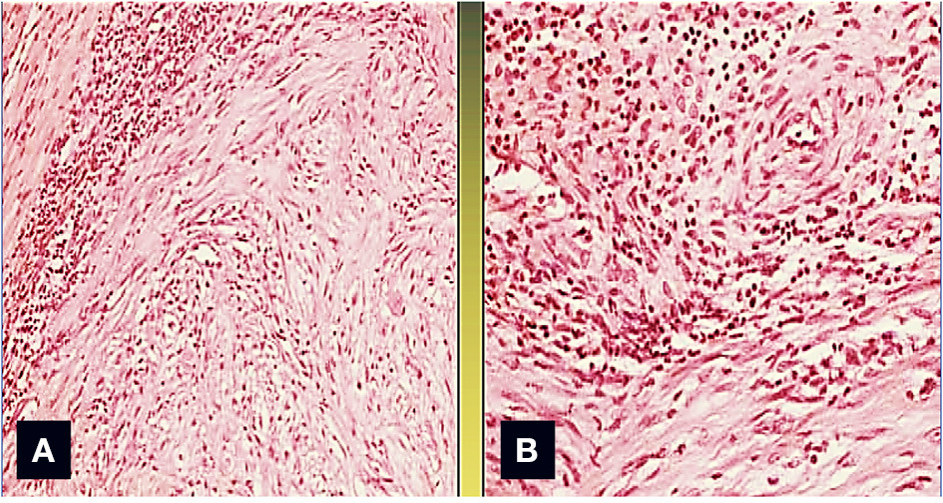
The area between the tumor and the normal myocardium is characterized by a mixed inflammatory infiltrate composed of polymorphonuclear leukocytes, lymphocytes, monocytes, and plasma cells (Col. Hem-Eos, original magnification ×40: **A,B**).

**Figure 3:**
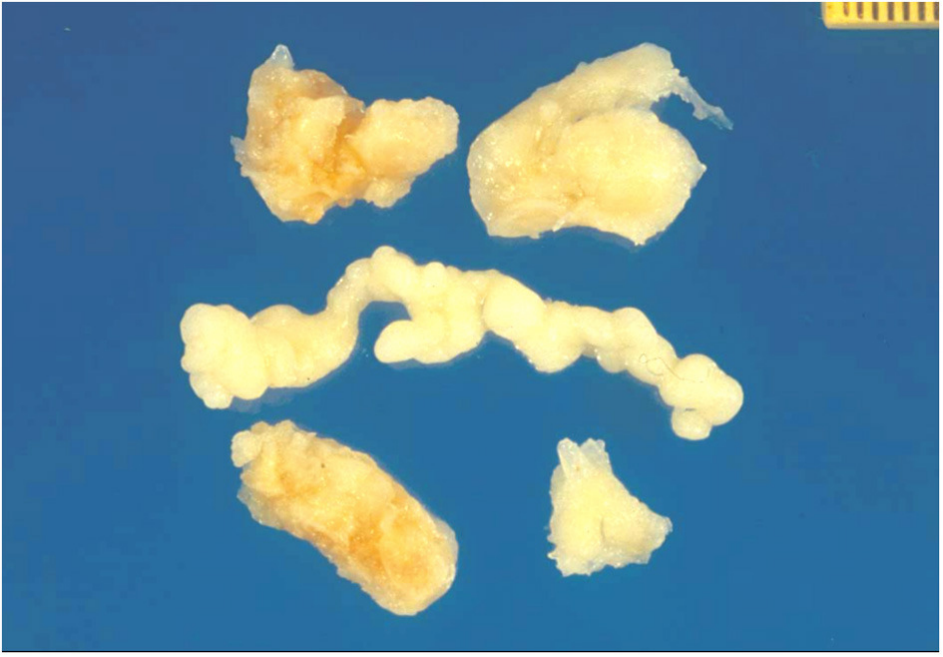
Gross view: lobulated and polypoid tumor measuring in cm 4 × 0.3 × 0.2, of whitish color, and of elastic consistency. Compact tissue with focal mucoid degeneration was observed to cut.

**Figure 4:**
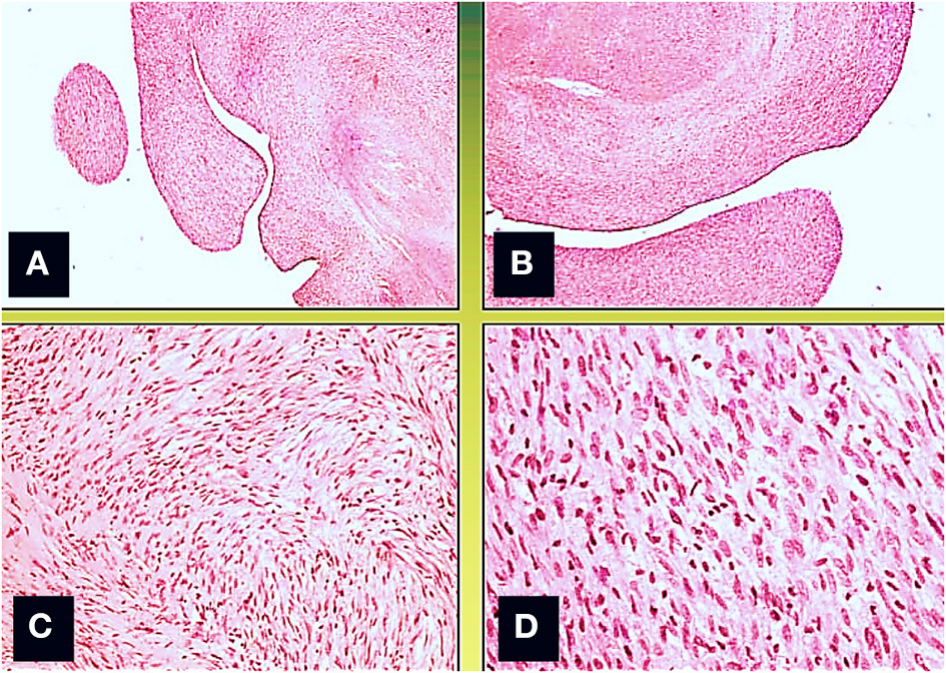
Microscopic view; the neoformation, localized predominantly in the subendocardium, has a polypoid aspect **(A,B)**, and it consists of monomorphic spindle cells, without atypia, dense proliferation, arranged both in parallel bundles **(B)** and storiform pattern **(C)**. Coagulative necrosis was observed in the central part of the tumor (Col. Hem-Eos, original magnification ×10: **A,B**; ×40: **C,D**).

**Figure 5:**
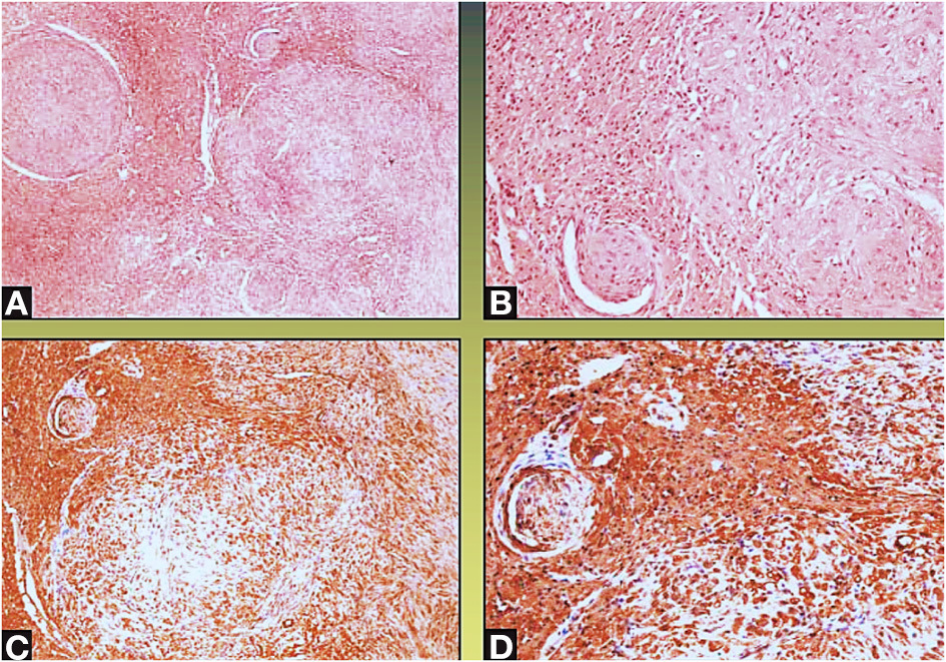
Neoplastic spindle cells infiltrate the small intramural coronary vessels extrinsically, with subsequent flow obstruction and ischemic necrosis of the tributary myocardium (Col Hem-Eos: **A,B**; actin: **C,D**; original magnification ×10: **A,D**; ×40: **B,C**).

**Figure 6:**
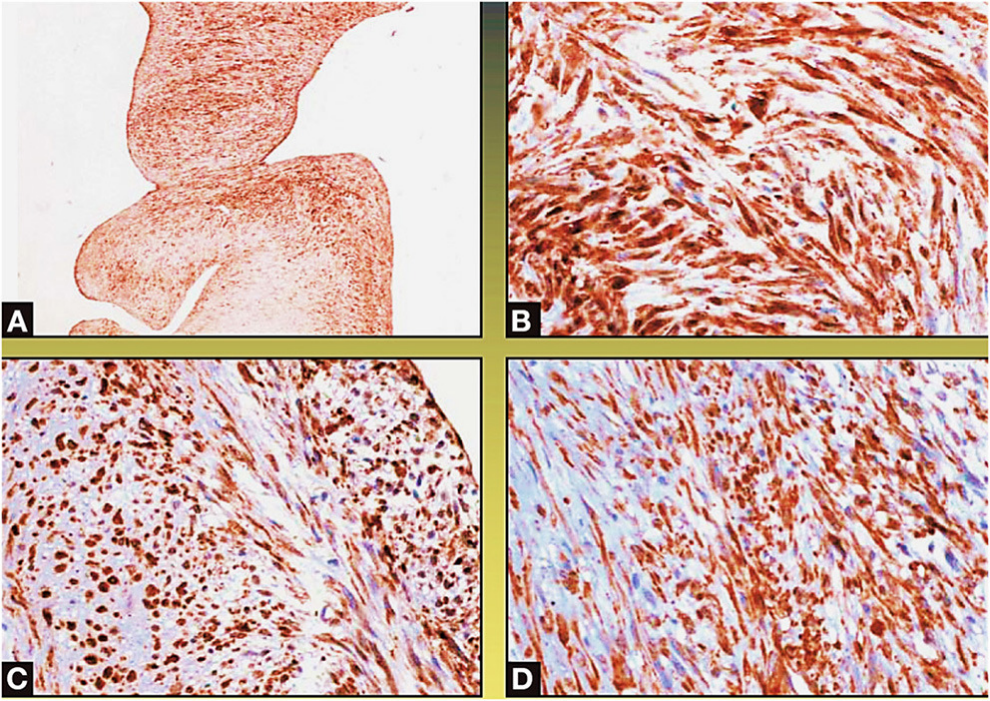
Spindle cells showing positivity for the actin—muscle specific **(A,B)** and vimentin stain **(C,D)** (original magnification ×10: **A**; ×40: **C,D**; ×100: **B**).

**Figure 7:**
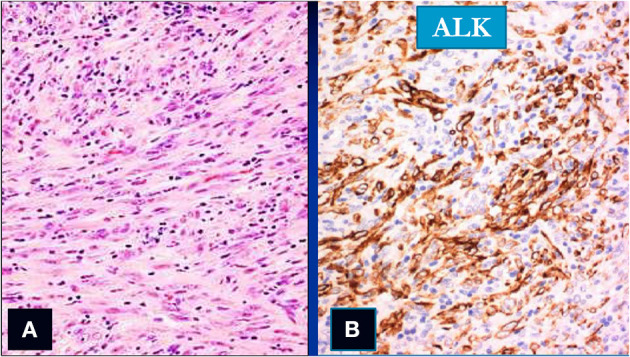
Spindle cells (col Hem-Eos: **A**) showing positivity for the ALK stain **(B)** (original magnification ×10).

A surgical exploration revealed the presence of a polypoid, non-encapsulated, and lobulated neoplasm measuring 4 × 0.3 × 0.2 cm (Figure 2), with hard elastic consistency, smooth external surface, and foci of mucoid degeneration (Figure 3) surgically removed by exeresis through transaortic access, obtaining samples for the histological, histochemical, and immunohistochemical analysis. The histological exams, performed with hematoxylin–eosin, Masson's trichrome, Alcian blue PAS, and Weigert and Van-Gieson stain, allowed observing the microscopic structure of the neoplastic mass, consisting of spindle monomorphic cells, arranged in parallel bundles and a storiform pattern (Figure 4), and conspicuous inflammatory components with a population of lymphocytes and granulocytes. The inflammatory component was located between the neoplasm and the healthy myocardial tissue. The spindle cells infiltrated the small intramural coronary vessels with consequent obstruction and ischemic necrosis of the tributary myocardium (Figure 5). The immunohistochemical analysis was performed through subsequent antibodies: anti-vimentin, anti-actina, anti-ALK, anti-CD8, and anti-CD3. Spindle cells showed positivity for anti-vimentin, anti-ASMA, anti-muscle specific actin (Figure 6), and anti-ALK (Figure 7); the inflammatory component resulted positive for anti-CD3, anti-CD 8, anti-CD20, anti-kappa, and lambda chains and anti-CD68 antibodies.

Cytokeratin was negative. These findings corresponded to the literature data based on macroscopic examination of the mass, distribution, conformation, and type of cells, as well as positivity for anti-vimentin and anti-muscle-specific actin antibodies (9, 10).

The healthcare professionals diagnosed a cardiac IMT with cerebral embolism.

## Differential Diagnosis

The location of the uncommon tumor in the heart made clinical and histopathological diagnosis difficult secondary to its clinical and morphological analogies with other cancerous masses. In this case, the ventricular localization, observed through radiological exams, required a differential diagnosis with fibroma and rhabdomyoma, the presence of brain embolism with sarcoma, and its morphology with fibroma. The histological exams are fundamental in the differential diagnosis from fibroma: this last, in fact, consists of monomorphic spindle cells, characterized by nuclei devoid of nucleoli and pale cytoplasm with entrapped myocytes, undulated elastic fibers, and rare lymphocytes; IMT, on the contrary, consists of large spindle cells with nuclei endowed of nucleoli, numerous acute and chronic inflammatory cells, and a dense vascular network.

The immunohistochemical analysis facilitated its diagnosis secondary to the positivity of IMT cells for the expression of high muscle-specific actin and negativity for the expression of cytokeratin, CD34, S100, and p53 proteins. It has been difficult to get to a true diagnosis in our clinical case. Clinical manifestation, in fact, only consisted of neurological symptoms: right motor delay, state of slumber and positive Babinski sign, aphasia, right upper limb hypertonus, miosis, and generalized tonic–clonic seizures. No alterations in hemodynamic parameters were highlighted; electrocardiogram showed the electrical activity of the heart within normal limits. Microbiological exams became necessary, since the clinical presentation mimicked the symptoms and signs of encephalitis. Neurological symptoms of this clinical case might be attributed also to a primitive cerebral cancer, such as astrocytoma or embolic stroke. In these last cases, radiographic imaging allowed performing an accurate differential diagnosis.

## Discussion

The cardiac tumors are subdivided into two categories: primary and secondary malignancies. The primary tumors (this definition including benign and malignant tumor) are very rare (11, 12): the autopsy incidence of this tumor is about 0.001–0.03% (13).

The inflammatory myofibroblastic tumor represents a rare neoplasm with a not entirely determined anatomopathological identity and uncertain biological behavior. It affects predominantly children and young adults, with localization in soft tissues and various organs. Histologically, this type of neoplasm consists of myofibroblasts, inflammatory cells, namely, plasma cells, lymphocytes, eosinophils, and fibromyxoid stroma. In our case, the IMT localization was at the level of the heart ventricular chamber. It may originate from atrial or ventricular walls, valves, or coronary vessels, at the heart level.

The cardiac IMT etiology is still unknown; there are many theories about it, such as fibroblastic inflammatory reaction due to traumatic or iatrogenic events; autoimmune disease, especially in cases of its association with vasculitis or venous thrombosis with antifibrinogen and anti-C3 antibody deposits in blood vessel walls; infections caused by *Escherichia coli*, Gram-positive cocci, *Klebsiella pneumoniae*, mycetes, or Epstein–Barr virus. Many classes of IMT, associated with chromosomal abnormalities, present an aggressive behavior, although this is a benign neoplasm. An important characteristic of IMT is the high tumor recurrence rate (about 25%), infiltration of adjacent structures, and distant metastases. The genetic and immunohistochemical studies allow discovering genetic modification, such as the 2p23 locus clonal rearrangements of the ALK gene, ALK gene fusion with specific proto-oncogenes (TPM3, TPM4) and clathrin heavy chain (CTLC) gene, and ALK gene overexpression. ALK-1 positivity was associated with a better prognosis in our case according to some studies (14); other authors support that there was no correlation between the two variables (6). At least 50% of IMT presents AlK-1 positivity; this proportion increases in the primary cardiac tumor: this immunochemical marker facilitates the diagnostic process (15, 16). Cytokeratin was negative; there was a weak immunopositivity of desmin. These results agree with literature data: most cases of IMT are negative for these markers (5).

The American Academy of Pediatrics provided guidelines for overall pediatric cancer recommending a multidisciplinary approach (17).

There are no specific guidelines about IMT; the European Society of Cardiology suggests a radiological diagnostic approach with echocardiography or real-time three-dimensional echocardiography to get information about the tumor blood supply or composition, relationship to nearby structures, and complementary imaging exams, such as CT, CMR, and PET, to detect its extension and resectability.

The echocardiography, CT, and CMR performed in our clinical case provided a valid aid in the diagnosis, but nevertheless, the histopathological examination resulted, clearly, fundamental to a definite diagnosis.

Regarding the therapeutic strategy, the National Cancer Institute summarizes five types of approach: watchful waiting, chemotherapy, surgery, radiation therapy, and target therapy (8). In case of high recurrence risk, such as our case (25%), a surgical approach would seem beneficial, whenever possible. Surgical resection still remains the main treatment (6). The role of chemotherapy and radiotherapy is still doubtful (18). Recent studies showed that the chemotherapy could improve the outcome of patients with an advanced tumoral disease; the role of ALK inhibitors in the case of ALK positivity is still an object of a study (19).

The therapeutic protocol remains uncertain because of the lack of clinical trials concerning this rare neoplasm (19).

## Conclusions

New studies are encouraged to better define IMT behavior and draw up guidelines confirming the crucial role of multidisciplinary approach and treatment protocol, selected on the basis of the characteristics of the tumors, in the case of this rare type of cancer.

## Data Availability Statement

The raw data supporting the conclusions of this article will be made available by the authors, without undue reservation.

## Ethics Statement

Ethical review and approval was not required for the study on human participants in accordance with the local legislation and institutional requirements. Written informed consent from the participants' legal guardian/next of kin was not required to participate in this study in accordance with the national legislation and the institutional requirements.

## Author Contributions

CB, GP, PD, EP, AA, and SZ performed the literature research and comparing literature data with case report clinical funding. EM and GD performed the immunohistological analysis. All authors contributed to the article and approved the submitted version.

## Conflict of Interest

The authors declare that the research was conducted in the absence of any commercial or financial relationships that could be construed as a potential conflict of interest.

## Publisher's Note

All claims expressed in this article are solely those of the authors and do not necessarily represent those of their affiliated organizations, or those of the publisher, the editors and the reviewers. Any product that may be evaluated in this article, or claim that may be made by its manufacturer, is not guaranteed or endorsed by the publisher.

## Abbreviations

ECG: electrocardiogram
IMT: inflammatory myofibroblastic tumor
CT: computed tomography
MR: magnetic resonance.
